# Clinical significance and risk factors of International Society of Urological Pathology (ISUP) grade upgrading in prostate cancer patients undergoing robot-assisted radical prostatectomy

**DOI:** 10.1186/s12885-021-08248-y

**Published:** 2021-05-04

**Authors:** Yuta Takeshima, Yuta Yamada, Taro Teshima, Tetsuya Fujimura, Shigenori Kakutani, Yuji Hakozaki, Naoki Kimura, Yoshiyuki Akiyama, Yusuke Sato, Taketo Kawai, Daisuke Yamada, Haruki Kume

**Affiliations:** 1 Division of Innovative Cancer Therapy, The Advanced Clinical Research Center, The Institute of Medical Science, The University of Tokyo, Minato-Ku, Tokyo, Japan; 2 Department of Urology, Graduate School of Medicine, The University of Tokyo, Hongo7-3-1, Bunkyo-Ku, Tokyo, Japan; 3 Department of Urology, Jichi Medical University, Shimotsuke-shi, Tochigi Japan; 4 Department of Urology, Chiba Tokushukai Hospital, Funabashi-shi, Chiba Japan

**Keywords:** Prostate cancer, Robot-assisted radical prostatectomy, Pathological upgrading, Biochemical recurrence

## Abstract

**Background:**

The objective of this study is to investigate the clinical significance and risk factors of upgrading in the International Society of Urological Pathology (ISUP) Grade Group System in men undergoing robot-assisted radical prostatectomy (RARP) for prostate cancer.

**Methods:**

A total of 583 patients diagnosed with prostate cancer by systematic biopsy were treated with RARP without neoadjuvant therapy from November 2011 to December 2018. Clinicopathological data were obtained from our clinical records. ISUP grade upgrading (IGU) was defined as ‘ISUP grade in prostatectomy specimen determined to be higher than that in the biopsy specimen’. Clinicopathological factors, including age, PSA, prostate volume at biopsy (PV), PSA density, clinical stage, body mass index (BMI), interval from biopsy to prostatectomy, maximum percentage of cancer involvement per core (%CI), total number of biopsy cores, percentage of cancer positive biopsy cores (%PC), and sampling density were analyzed to detect potential risk factors of IGU. Biochemical recurrence (BCR) rates were calculated to analyze the effect of IGU on cancer prognosis.

**Results:**

In univariate analysis, BMI was a positive predictor of IGU, while %CI, %PC, and sampling density were negative predictors of IGU. BMI and %PC were statistically significant predictors of IGU in multivariate analysis. For cases diagnosed as ISUP grade group 2 or higher at biopsy, there was a significant difference in BCR rates between cases with and without IGU.

**Conclusions:**

The results from our cohort showed that elements of both high-grade cancer risk (such as BMI) and sampling efficiency (such as %PC) contribute to IGU. Excluding cases diagnosed as ISUP grade group 1 at biopsy, BCR-free rates were significantly worse in cases with IGU, highlighting the need for more accurate pathological diagnosis at biopsy.

**Supplementary Information:**

The online version contains supplementary material available at 10.1186/s12885-021-08248-y.

## Background

Prostate cancer is the most common malignancy in men and the second leading cause of cancer death in the United States [1]. Pathologically, PCa is frequently comprised of multiple tumor grades, defined by Gleason score (GS). GS is one of the strongest predictors of disease outcome, the cornerstone in risk stratification of prostate cancer, and as such is usually the pre-eminent factor in counseling early-stage patients for treatment options [2]. Since 2014, GS has been reclassified by the International Society of Urological Pathology (ISUP) into the ISUP Grade Group system [3]. This new grouping system has also been shown to be significant predictors of cancer prognosis such as biochemical recurrence (BCR)-free survival and cancer-specific mortality [4, 5].

ISUP grade determined by prostate biopsy has assumed even greater importance in recent years with a relative increase in patients undergoing therapy other than radical prostatectomy, namely radiation therapy and active surveillance, where the only tissue analyzed is from the biopsy. However, ISUP grade diagnosed from the biopsy samples frequently disagree with the results from the RP specimen, with reported rates varying from 14 to 72% [6, 7]. According to one large-scale analysis, the frequency of GS upgrading and downgrading in a cohort of 7643 were reported as 36.3 and 12.0%, respectively, representing a stronger tendency for biopsies to underestimate rather than overestimate the true GS [8]. Not surprisingly, GS upgrading has been associated with worse outcomes, and survival curves of BCR have been reported to resemble that of the higher grade more closely [9]. Thus, the identification of pre-operative factors that predict GS upgrading is an area of strong interest. However, the majority of the literature predates the introduction of ISUP grade groups, and updated analysis may be of clinical interest.

The purpose of this study is to determine clinical and pathological predictors of ISUP grade upgrading (IGU) and to analyze the effect of IGU on BCR rates. Such findings may aid us to better assess the true risk of prostate cancer and counsel patients on treatment and prognosis.

## Methods

### Patient characteristics

A total of 6 hundred and 30 patients with prostate cancer were treated with robot-assisted radical prostatectomy (RARP) from November 2011 to December 2018 at the University of Tokyo Hospital. The da Vinci surgical robot system (da Vinci-S or Xi®: Intuitive Surgical Incorporation, Sunnyvale, CA) was used in performing RARP. As described in our previous studies, RARP was performed using the transperitoneal, 6 port technique [10, 11]. Sixteen patients were treated with androgen deprivation therapy prior to RARP and were excluded from this study. Additionally, 31 patients had been diagnosed by a biopsy protocol including MRI-targeted biopsy and were also excluded. A total of 583 patients were eligible for the final analysis. Clinicopathological data including age, body mass index (BMI), initial prostate specific antigen (PSA), prostate volume on ultrasound (PV), clinical T stage, interval time from biopsy to RARP, pre-operative ISUP grade, post-operative ISUP grade, percentage of cancer positive cores in biopsy (%PC), maximum % of cancer involvement in a single core (%CI), and ‘sampling density’ were obtained from our clinical records. ‘Sampling density’ was originally coined by Chun et al. as the ratio between total gland volume and number of cores sampled (ml/core), and it has been identified as a predictor of prostate cancer detection on repeat biopsy [12]. By this definition, a ‘higher’ sampling density would represent a smaller number of cores per prostate volume, which seems counterintuitive in the context of the present study on whether sampling efficiency contributes to IGU. Therefore, we used its reciprocal, or number of biopsy cores per prostate volume, as our definition of ‘sampling density’. IGU was defined as ‘Higher ISUP grade in the prostatectomy specimen compared to that of biopsy specimen’. Clinical and pathological factors of IGU were investigated. Clinical and pathological stage of prostate cancer was determined using the American Joint Committee on Cancer TNM staging system (8th edition). Routine follow-ups were conducted at 2 weeks, 1, 3, 6, 12 months post-discharge, and on a 6–12 month cycle thereafter.

All patients provided a written informed consent. This study was in accordance with the Helsinki declaration and was approved by the institutional review board of the Tokyo University Hospital (approval no. 3124).

### Biopsy procedure

Transrectal/transperineal ultrasound-guided prostate biopsy was performed by experienced urologists in all cases, with number of cores varying by institution of referral (median 12 cores). The patients that were evaluated received only systematic biopsy, and those in whom MRI-targeted biopsy cores were retrieved were excluded from this study.

### Pathological analysis

All biopsy specimens were routinely reported as the consensus opinion of two experienced pathologists. In cases of referral, the biopsy specimen was obtained from the referring institution and re-analyzed at the University of Tokyo Hospital. Biopsy core specimens were routinely assessed for ISUP grade, percentage of tumor involvement in each core, and location.

Prostatectomy specimens were similarly reported as the consensus opinion of two experienced pathologists. Specimens were examined as whole-mount preparations, and were routinely assessed for ISUP grade, surgical margin positivity, extracapsular extension, perineural invasion, seminal vesicle invasion, bladder neck invasion, multifocality, diameter of each focal tumor, and zonal location. All pathological analyses were conducted in accordance with the 2005 update and 2014 ISUP consensus [3].

### Statistical analyses

Wilcoxon rank-sum tests were used to compare continuous values between 2 groups. Categorical values were analyzed by Pearson’s chi-square test (χ^2^) and Fisher exact test. Univariate and multivariate analyses using logistic regression models were performed to evaluate which clinical parameters were associated with IGU. Kaplan-Meier curves were drawn for BCR-free survival, and the log-rank test was performed to compare survival between groups. A *P*-value of < 0.05 was considered statistically significant. All statistical analyses were performed using JMP Pro® software, ver. 14.2 (SAS, Cary, NC, USA).

## Results

Demographics and pathology at RARP of the subjects are shown in Table 1. A total of 224 men had IGU (38.4%), while 78 (13.4%) had ISUP grade downgrading. Higher BMI and lower clinical T stage were associated with IGU (*P* = 0.012 and *P* = 0.024, respectively). For parameters related to biopsy pathology, IGU was observed in patients with lower pre-operative ISUP grade, lower %IC, lower %PC, and sampling density. Age, PSA, PV, PSAD, the time interval from biopsy to RARP, and the total number of biopsy cores did not affect IGU.

**Table 1:** Patient Demographics

			All cases	Upgrading	No upgrading	*P* value
No. of patients			583 (100%)	224 (38.4%)	359 (61.6%)	
Age		years (median, IQ)	66.7 (64.0–71.0)	67.2 (64.0–71.0)	66.4 (63.0–71.0)	0.198
PSA		ng/ml (median, IQ)	9.7 (5.5–11.0)	9.3 (5.5–10.4)	9.9 (5.5–11.3)	0.609
PV		ml (median, IQ)	32.0 (21.0–38.0)	32.7 (22.0–39.0)	31.5 (21.0–37.8)	0.065
PSAD		ng/ml^2^ (median, IQ)	0.35 (0.18–0.41)	0.32 (0.18–0.39)	0.37 (0.18–0.43)	0.275
BMI		kg/m2 (median, IQ)	23.9 (22.0–25.5)	24.4 (22.4–25.8)	23.6 (21.9–25.2)	0.012**
cT stage	cT1	no. of patients (% of total)	461 (79.2%)	189 (84.4%)	272 (76.0%)	0.024*
	cT2a	no. of patients (% of total)	60 (10.3%)	23 (10.3%)	37 (10.3%)	
	cT2b	no. of patients (% of total)	28 (4.8%)	9 (4.0%)	19 (5.3%)	
	cT2c	no. of patients (% of total)	27 (4.6%)	3 (1.3%)	24 (6.7%)	
	cT3–4	no. of patients (% of total)	6 (1.0%)	0 (0%)	6 (1.7%)	
ISUP grade^a^	1	no. of patients (% of total)	106 (18.2%)	89 (39.7%)	17 (4.7%)	< 0.001*
	2	no. of patients (% of total)	233 (40.0%)	89 (39.7%)	144 (40.1%)	
	3	no. of patients (% of total)	97 (16.6%)	21 (9.4%)	76 (21.2%)	
	4	no. of patients (% of total)	89 (15.3%)	25 (11.2%)	64 (17.8%)	
	5	no. of patients (% of total)	58 (9.9%)		58 (16.2%)	
Biopsy-prostatectomy interval		days (median, IQ)	133.6 (85–140)	131.2 (86–139)	135.2 (85–145)	0.570
%IC		%(median, IQ)	46.0 (21.5–70.0)	40.8 (16.8–60.0)	49.3 (30–70.0)	< 0.001*
Total no. of cores		(median, IQ)	12.0 (9–12)	11.8 (9–12)	12.2 (9–13)	0.441
%PC		%(median, IQ)	30.0 (12.5–41.7)	25.8 (11.1–36.9)	32.7 (15.4–44.4)	< 0.001*
Sampling density		core/ml (median, IQ)	0.44 (0.30–0.55)	0.42 (0.29–0.52)	0.46 (0.30–0.57)	0.043*

^*^ statistically significant, ^a^: ISUP grade as diagnosed by prostate biopsy

*Abbreviations*: *IQ* interquartile, *PSA* prostate specific antigen, *PV* prostate volume, *PSAD* PSA density, *BMI* body mass index, *cT stage* clinical T stage, *ISUP* International Society of Urologic Pathologists, *%IC* maximum percentage of cancer involvement per core, *%PC* percentage of positive cores

In multivariate analyses, BMI and %PC remained statistically significant predictors (Table 2).

**Table 2:** Univariate and multivariate analysis for risk factors of ISUP grade upgrading at robot-assisted radical prostatectomy

	Univariate				Multivariate			
	OR	95%CI	95%CI	*P* value	OR	95%CI	95%CI	*P* value
Age	1.023	0.994	1.052	0.120				
PSA	0.988	0.966	1.012	0.331				
PV	1.005	0.995	1.015	0.349				
PSAD	0.563	0.300	1.059	0.075				
BMI	1.097	1.033	1.165	0.026*	1.101	1.034	1.173	0.003*
Biopsy-prostatectomy interval	1.000	0.998	1.001	0.684				
%IC	0.989	0.983	0.995	< 0.001*	0.996	0.988	1.003	0.261
Total no. of cores	0.976	0.931	1.022	0.298				
%PC	0.982	0.974	0.991	< 0.001*	0.985	0.975	0.996	0.006*
Sampling density	0.398	0.171	0.928	0.033*	0.466	0.197	1.106	0.068

* statistically significant

*Abbreviations*: *OR* odds ratio for unit increase, *CI* confidence interval, *PSA* prostate specific antigen, *PV* prostate volume, *PSAD* PSA density, *BM*I body mass index, *%IC* maximum percentage of cancer involvement per core, *%PC* percentage of positive cores

**Fig. 1:**
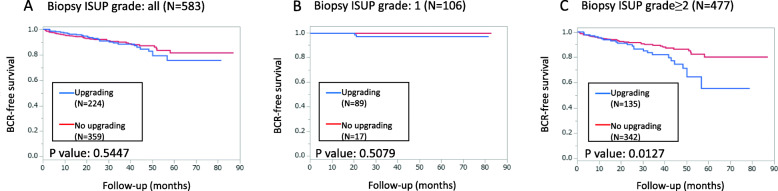
Biochemical recurrence-free survival of patients stratified by pre-operative ISUP grade. **a** All patients **b** Patients with preoperative ISUP grade 1 **c** Patients with preoperative ISUP grade 2 or higher. Comparisons of Kaplan-Meier curves for patients with and without ISUP grade upgrading are given for all groups. A statistically significant difference was found in group C (Log-rank test: P value = 0.013)

There was no significant impact of IGU on PSA free survival in the study subjects overall (Fig. 1a, *P* value = 0.5447), at a median follow-up of 31.3 months (interquartile 18.9–47.9). However, when we excluded patients with ISUP grade 1 at biopsy (Fig. 1b), patients with IGU exhibited significantly lower BCR-free survival (Fig. 1c, *P* value = 0.013) at a median follow-up of 29.4 months (interquartile 17.3–45.8). We further stratified this group by surgical margin status and found that patients with IGU exhibited significantly lower BCR-free survival in the negative surgical margin group (Suppl. Fig. 1A), but not in the positive surgical margin group (Suppl. Fig. 1B).

## Discussion

Discordance in pathological grades between biopsy and prostatectomy specimens has been regarded as a ‘misdiagnosis’, possibly leading to a worse prognosis due to inappropriate selection of therapeutic options. The prognostic impact of GS upgrading upon radical prostatectomy has been reported in a few studies [9, 13–15]. While the individual methods have varied, mostly on the configuration of subgroups analyzed for comparison, the overall evidence shows that IGU does indeed lead to a decline in BCR-free rates. In our cohort, there was no statistical difference between cases with and without IGU overall, but when restricted to cases with a pre-operative ISUP grade of ≥2, BCR-free rates were significantly lower in cases with IGU than those without. This is most likely due to the fact that BCR-free rates in the cases with a pre-operative ISUP grade of 1 were extremely high regardless of the presence or absence of IGU. Positive surgical margin, which implies the presence of unexcised tumors, has understandably been identified as a strong marker of BCR [16]. We further stratified the ISUP grade ≥ 2 group by positive/negative surgical margin and found that the decline of BCR-free rates in the IGU group was retained in the negative surgical margin group. Our results reinforce the notion that prevention of IGU would lead to a better evaluation of cancer prognosis and would benefit clinical decision-making, at least for cases with a pre-operative ISUP grade of 2 or higher.

In order to gain insight into how urologists can prevent GS upgrading, previous studies have analyzed and identified multiple clinical parameters as independent predictors. Of these, factors such as higher age, PSA, PSAD, BMI, %CI, and %PC, smaller PV, and longer interval between biopsy and prostatectomy have been identified as significant risk factors of pathological upgrading [8, 17–22]. These studies argue, understandably, that the inherent risk of higher-grade disease derived from clinical parameters would lead to a higher probability of the post-operative pathology exhibiting a higher grade than diagnosed by biopsy. The risk of higher BMI, found to be an independent predictor of IGU in our cohort by multivariate analysis, is a prime example. Obesity has been reported to be a significant predictor of BCR and related to aggressive PCa, suggesting that prostate cancer cells in obese men grow faster [23]. Obesity is associated with high estrogen levels and low testosterone levels [24]. Since low testosterone level is associated with high grade prostate cancer, obesity may decrease testosterone levels leading to the development of aggressive PCa. This is further supported by a study that found low testosterone level to also be an independent risk factor of Gleason score upgrading [25]. BMI has also been linked to greater prostate volume and may have an influence on such factors as prostate volume or sampling density [26, 27]. However, in our cohort, correlation between BMI and prostate volume or BMI and sampling density did not show significant correlation.

Conversely, a few studies have identified predictors of upgrading related to the element of sampling efficiency. Factors such as larger PV, smaller number of total biopsy cores, lower %PC, and shorter biopsy core length were identified as risk factors of pathological upgrading [22, 28–32]. These studies suggest that improved sampling would lead to a more accurate evaluation of prostate cancer pathology, and thus to a decrease in GS upgrading. One proposed method for this is an increase in the total number of biopsy cores. The number of cores sampled in biopsy has gradually increased from the original sextant scheme to saturation biopsies of over 20 cores in selected cases. Understandably, an increase in total biopsy cores would prevent low-powered sampling and may therefore lead to a decrease in pathological upgrading upon prostatectomy. In the two studies which analyzed extended biopsy schemes, GS upgrading incidence was significantly lower in extended biopsy schemes when compared with traditional biopsy schemes [31, 32]. In our cohort, however, while a lower %PC was established as a significant predictor of IGU on multivariate analysis, the total number of cores was not significant. In addition, the average number of total cores for our cohort was 12.0 which is in line with a 12-point biopsy scheme recommended in current guidelines and can be considered sufficient in terms of sampling efficiency. These data suggest that further increase in the number of biopsy cores may not have led to improved sampling in our cohort. Recently, multiparametric magnetic resonance imaging (mpMRI)-targeted biopsy has been shown to decrease pathological upgrading [33–36]. Targeted biopsy may be an ideal strategy to improve the accuracy of biopsy pathology while avoiding adverse effects related to saturation biopsy. The percentage of patients receiving mpMRI-targeted biopsy at our institution has been increasing subsequent to this study, and we plan to evaluate its effect on IGU in the future.

Some limitations of the present study should be mentioned. First of all, the retrospective nature of the study design at a single institution may have biased the results. Secondly, the average observation period was relatively short, with a median of 31.3 months. As noted earlier, the high BCR-free rates of pre-operative ISUP grade 1 cases seem to have affected the BCR-free rates of the whole cohort strongly. Considering the long duration of prostate cancer progression, especially for low-grade cancer, a longer observation period may have led to different results on the analysis of BCR rates. Thirdly, the length of cores was not considered in the present study. As stated earlier, Reis et al. reported that shorter biopsy core length was an independent predictor of pathological upgrading, one of the examples of sampling efficiency affecting pathological examination [32]. We were unable to include this parameter in our analysis.

## Conclusions

Pathological grade on biopsy is frequently upgraded in prostatectomy specimens, possibly resulting in insufficient pre-operative evaluation of disease prognosis. Measures should be taken to prevent IGU for optimal input on clinical decision-making. Results from our cohort showed that elements of both high-grade cancer risk and sampling efficiency affected IGU.

## Supplementary Information

**Additional file 1: Suppl. Fig. 1** Biochemical recurrence-free survival of patients (pre-operative ISUP grade ≥ 2) stratified by surgical margin.

## Data Availability

The datasets used in the current study are not publicly available due to on-going clinical studies based on the same database but are available from the corresponding author on reasonable request.

## Abbreviations

BCR: Biochemical recurrence
BMI: Body mass index
GS: Gleason score
IGU: ISUP grade upgrading
ISUP: International Society of Urological Pathology
mpMRI: Multiparametric magnetic resonance imaging
PV: Prostate volume
PSA: Prostate specific antigen
PSAD: Prostate specific antigen density
RARP: Robot-assisted radical prostatectomy
%CI: Maximum percentage of cancer involvement in a single core
%PC: Percentage of cancer positive cores in biopsy
