# Supplementary material for: Clinical significance and risk factors of International Society of Urological Pathology (ISUP) grade upgrading in prostate cancer patients undergoing robot-assisted radical prostatectomy
Source: BMC Cancer. 2021 May 4;21:501. doi: 10.1186/s12885-021-08248-y (PMC8097801; doi:10.1186/s12885-021-08248-y)
Supplement: Supplementary file 1 — Additional file 1: Suppl. Fig. 1 Biochemical recurrence-free survival of patients (pre-operative ISUP grade ≥ 2) stratified by surgical margin. [file 12885_2021_8248_MOESM1_ESM.pptx]

## Slide 1
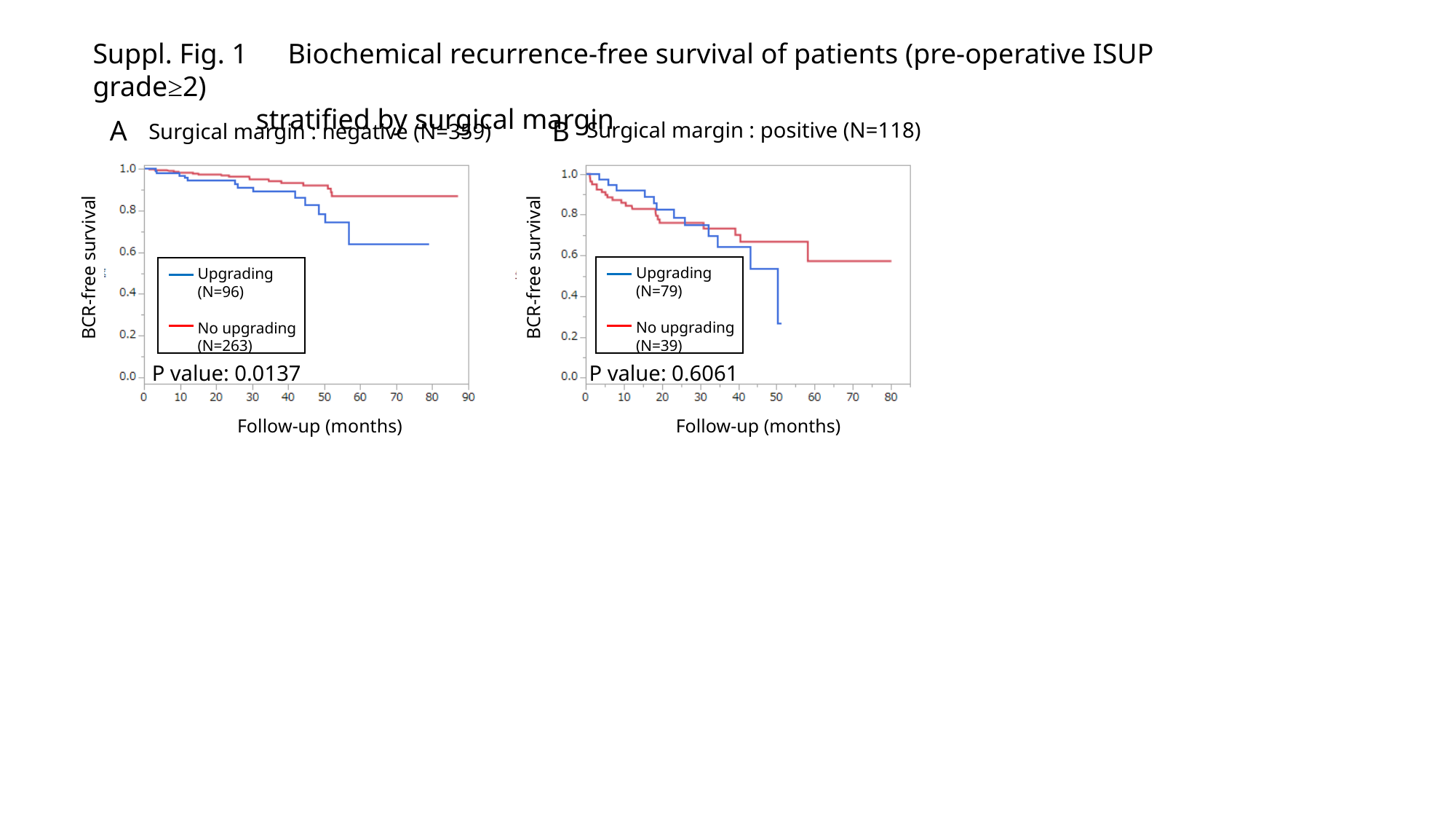

Suppl. Fig. 1　Biochemical recurrence-free survival of patients (pre-operative ISUP grade≥2)
 stratified by surgical margin
A
B
Surgical margin : positive (N=118)
Surgical margin : negative (N=359)
BCR-free survival
BCR-free survival
 Upgrading
 (N=79)
 No upgrading
 (N=39)
 Upgrading
 (N=96)
 No upgrading
 (N=263)
P value: 0.0137
P value: 0.6061
Follow-up (months)
Follow-up (months)
